# Anisotropic Wettability of Bioinspired Surface Characterized by Friction Force

**DOI:** 10.3390/biomimetics7030108

**Published:** 2022-08-08

**Authors:** Jinhong Zhang, Lijun Li, Peng Xu, Yifeng Lei, Qianlin Song, Junwei Liu, Yunhe Xiong, Sixing Yang, Yurong Zhang, Longjian Xue

**Affiliations:** 1 The Institute of Technological Science, School of Power and Mechanical Engineering, Wuhan University, South Donghu Road 8, Wuhan 430072, China; 2 Hubei Yangtze Memory Laboratories, Wuhan 430205, China; 3 Department of Mechanical Engineering, Shanxi Polytechnic College, Taiyuan 030006, China; 4 Urology Department, Renmin Hospital of Wuhan University, Zhangzhidong Road 99, Wuhan 430060, China

**Keywords:** bioinspired surface, wettability, contact angle, friction force, MPCP technique

## Abstract

Bioinspired surfaces with special wettabilities attract increasing attention due to their extensive applications in many fields. However, the characterizations of surface wettability by contact angle (CA) and sliding angle (SA) have clear drawbacks. Here, by using an array of triangular micropillars (ATM) prepared by soft lithography, the merits of measuring the friction force of a water droplet on ATM over measurements of CA and SA in characterizing the surface wettability are demonstrated. The CA and SA measurements show ignorable differences in the wettabilities of ATM in opposite directions (1.13%) and that with different periodic parameters under the elongation ranging from 0 to 70%. In contrast, the friction measurement reveals a difference of > 10% in opposite directions. Moreover, the friction force shows a strong dependence on the periodic parameters which is regulated by mechanical stretching. Increasing the elongation from 0 to 50% increases the static and kinetic friction force up to 23.0% and 22.9%, respectively. Moreover, the stick-slip pattern during kinetic friction can reveal the periodic features of the measured surface. The friction force measurement is a sensitive technique that could find applications in the characterization of surface wettabilities.

## 1. Introduction

Adapting to the living environment, plants and animals have evolved different surface features [1,2,3,4,5]. Quite often the surface features are anisotropic, containing either anisotropic micro- and nano-scale structures or anisotropic arrangements [6,7,8,9,10,11]. For instance, the direction-dependent, overlapping scales on butterfly wings enable water droplets on the surface to easily roll off, maintaining dry wings [6]. The aligned hairs, together with the nanogrooves on each hair, allow the water striders to effortlessly walk on water [7]. There are many more of these types of surfaces, on which water droplets show anisotropic wetting behaviors [8,9,10,11,12]. Inspired by the anisotropic surfaces found in nature, engineered surfaces with anisotropic wetting properties could contribute significantly to the fields, such as biomedicine, ship transportation, microfluidics, smart surfaces, and so on [12,13,14,15,16,17,18,19].

The static water contact angle (CA) and sliding angle (SA) have long been used to characterize the state of a water droplet on a solid surface [20,21,22,23]. The measured CA is usually an apparent CA, which is different from the inherent CA of an ideal smooth surface (CA_i_), as a surface always contains a certain roughness. The apparent CA is influenced by the coordination of the surface energy and roughness. In the following text, the mentioned CAs are apparent CAs, unless otherwise mentioned. The Wenzel model [24] has been used to describe the state when water can penetrate into the roughness on the surface. In contrast, the Cassie model [25] describes the surface where air cushions are maintained at the contacting interface. In both of the models, the surface roughness has a strong influence on the CA. Therefore, the precise characterization of the CA is of critical importance to understand the surface wettabilities. However, the water-contact-angle measurement has its inherent limitations, in that a one-pixel error in the definition of the baseline and/or the outline of the water droplet will cause a large deviation in the CA, especially on the superhydrophobic surface with a CA larger than 150° [20,26].

Meanwhile, the measurement of the SA also has its difficulties when the water droplet keeps sticking onto the surface when the surface is turned upside down, which is known as the rose petal effect [27,28]. On the surfaces with a high adhesion to water droplets, the measurements of the CA and SA may have difficulties in distinguishing the surfaces with subtle differences. For instance, on the surface composed of a polydimethylsiloxane (PDMS) triangular-micropillar array, with the period ranging from 40 to 50 µm, a negligible difference in the SAs (ΔSA = 3°) in opposite directions can be found [16]. Similarly, on the microstrip surfaces, with the air fraction ranging from 0.20 to 0.50, the water droplets kept sticking to the surface when the surface was turned upside down along the direction perpendicular to the microstrips [17]. In both cases, there is no difference in the SA along certain directions, even though the surface structures are distinctly different [16,17]. It clearly suggests that the SA measurement cannot reveal the structural difference of these examined surfaces and what the extent of the difference could be [16,17].

As a complementary technique to the CA and SA measurements, we have proposed a technique termed the monitoring of the position of capillary’s projection (MPCP), to characterize the friction force (F) of a water droplet on superhydrophobic surfaces [17]. When measuring F, the displacement–time curve could also reveal the structural information of the tested surfaces [17,29,30]. For instance, when measuring a fresh lotus leaf, the F_f_ suddenly changed when the droplet passed over the veins in the leaf. It can also measure the F_f_ on a butterfly wing in the directions towards the body center. It is worth mentioning that the water droplets usually stick firmly to the surface in the direction towards the body center. Therefore, MPCP is capable of offering a deeper insight into the liquid–solid interactions.

Here, we fabricate an array of triangular micropillars (ATM) and test the friction of a droplet on its surface in comparison with the traditional CA measurements. The CA remains the same when the period of ATM is regulated by mechanical stretching. In contrast, the MPCP reveals a clear difference in the friction forces in opposite directions on the ATM and that of the ATM with different structure parameters. Moreover, the detailed features of the kinetic friction process can also reveal the structure features of the ATM surface. Thus, the results here offer us the chance to better understand bioinspired surfaces with special wettabilities. 

## 2. Materials and Methods

### 2.1. Materials and Preparation

The PDMS elastomer kit (Sylgard 184) was obtained from Dow Corning (Midland, MI, USA). A PDMS precursor was prepared by uniformly mixing the prepolymer and cross-linker in a ratio of 10:1 (by weight). After degassing, the PDMS precursor was poured onto the silicon template with triangular microhole array, which was fabricated by standard photolithography [31]. After degassing for 10 min in a vacuum, the sample was cured at 90 °C for 1 h. The ATM was obtained by demolding from the silicon template. 

### 2.2. Surface Wettability

The water CA and CA hysteresis were measured by droplet shape analysis (OCA25, Dataphysics, Hamburg, Germany) at room temperature. The volume of the water droplet for CA measurement was 3 μL. The CA hysteresis was evaluated by continuously delivering water up to a volume of 15 μL at a dosing rate of 1 μL/s and retreated at the same speed. The CA and CA hysteresis were measured at five different positions on the ATM surfaces, and the mean values were calculated.

The liquid–solid friction test was conducted on a home-made device, as reported in our previous work [17]. In a typical measurement, the ATM sample was stuck onto the motor stage by a double-sided sticky tape. A 6 µL water drop was delivered onto the sample surface, using a stainless-steel capillary. The length, inner, and outer diameter of the capillary are 50, 0.11, and 0.21 mm, respectively. Before the movement of the stage, the capillary end was positioned at the droplet center. During the test, the movement of the stage drove the relative displacement of the sample to the droplet at a constant speed of 0.2 mm/s, while the projection of the capillary was monitored and recorded by a high-speed camera. The deviation of the capillary to its original position (*D*) was converted into friction force (*F*) according to equation (1):

*F* = k*D* (1)

where k is the spring constant of the capillary [17]. The average values of friction forces were acquired by measuring the ATM surfaces at no less than five different positions.

### 2.3. Surface Morphology

The microstructures of the samples were characterized by an optical microscopy (ECLIPSE Ci-L, Nikon, Tokyo, Japan) and a white light interference 3D profiler (NewView^TM^9000, ZYGO, Middlefield, CA, USA). The contact states of the water droplet on ATM were investigated on an inverted optic microscopy (ECLIPSE MA100N, Nikon, Tokyo, Japan).

## 3. Results and Discussion

### 3.1. Fabrication of ATM

**Figure 1:**
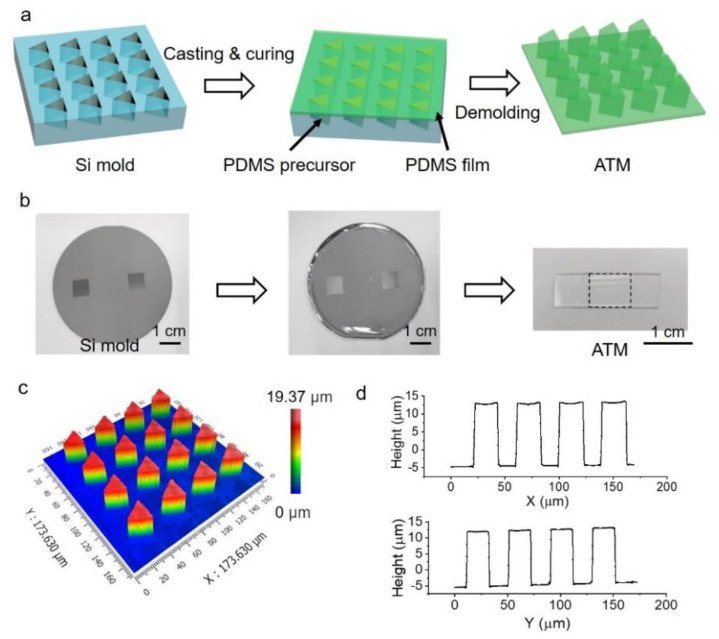
Fabrication of PDMS array of triangular micropillars (ATM). (**a**) Schematic illustration of the fabrication process; (**b**) Optical images of silicon mold, the spreading of PDMS precursor on silicon mold and the resulted sample, the dashed square indicates the location of ATM; (**c**) 3D morphology of ATM; (**d**) Typical profile of ATM along orthogonal directions.

The ATM was successfully constructed by a standard soft lithography procedure. PDMS precursor was filled into the triangular microholes on a silicon mold, followed by curing at 90 °C for 1 h (Figure 1a). Generally, 2 mL of PDMS precursor was spread onto the whole silicon mold, which resulted in a thickness of ~1.2 mm. After peeling off from the mold, the sample was cut into a strip (~1 cm in width and ~5 cm in length) with the ATM in the center region for the convenience of the following studies (Figure 1b). The resultant ATM is an array of micropillars with a height of ~20 μm, period of 40 μm, and a triangular cross-section with edge length of ~23 μm, faithfully copying the structures of the silicon mold (Figure 1c,d). The top of the micropillars is rather smooth, with a roughness of 1.3 ± 0.2 nm.

**Figure 2:**
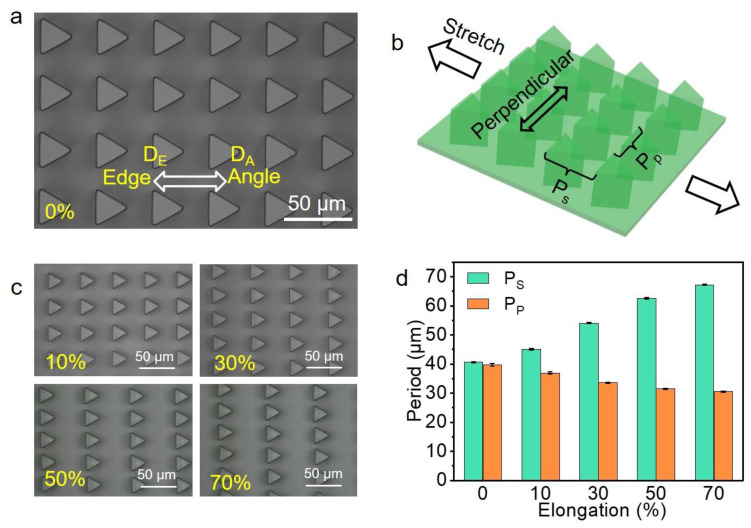
Surface topography of ATM. (**a**) Typical optical image of the resulted ATM with the definitions of directions of D_A_ and D_E_; (**b**) The definition of stretching and perpendicular directions with the corresponding period of P_s_ and P_p_ indicated; (**c**) Typical optical images at various stretching ratios; (**d**) The resulted P_s_ and P_p_ at various stretching ratios, each data point in (**d**) represents the mean value of at least 10 measurements. Standard deviations are indicated by error bars.

The periods of the micropillars in the ATM can be easily regulated by mechanical stress in the supporting layer. Due to the triangular shape of the micropillars, we therefore define the direction pointing to the angle of the triangular micropillar as the direction of angle (D_A_), and the opposite direction towards the bottom edge of the triangle as the direction of edge (D_E_) (Figure 2a). The stretching, in the direction of D_A_/D_E_, will thus change the period along the stretching direction (P_s_) and the period perpendicular to the stretching direction (P_p_) (Figure 2b). The as-prepared ATM has P_s_ = P_p_ = 40.62 ± 0.49 μm. Due to the Poisson’s ratio effect, the stretching increased P_s_ and decreased P_p_ (Figure 2c). For instance, the stretching to 30% increased P_s_ to 54.11 ± 0.19 μm and decreased P_p_ to 33.60 ± 0.15 μm (Figure 2d). The stretching to 70% further increased P_s_ to 67.25 ± 0.26 μm and decreased P_p_ to 30.51 ± 0.16 μm. The relaxation of the stress would recover the original structural parameters, due to the elasticity of PDMS [16].

### 3.2. Characterization of Wetting Property of ATMs 

**Figure 3:**
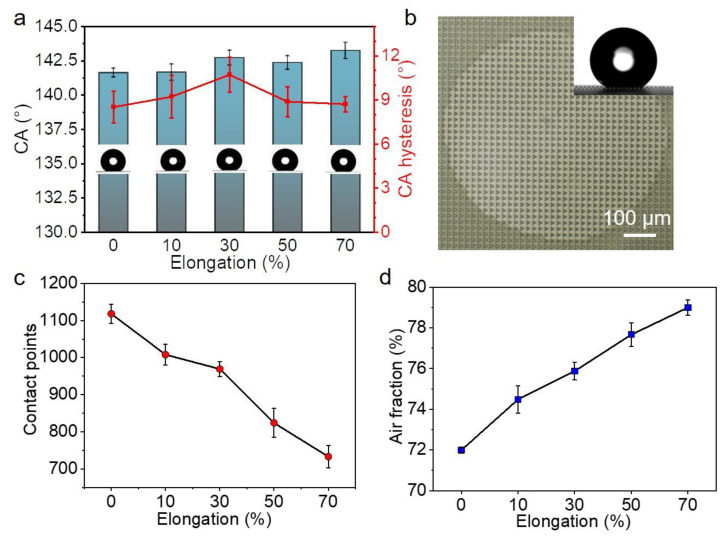
Wettability characterization by water contact angle (CA). (**a**) CA and CA hysteresis of ATM at various stretching ratios; (**b**) Typical optical image of the contact interface, the inset is a typical image of CA measurement; (**c**,**d**) The dependence of (**c**) contact points and (**d**) air fraction on the stretching ratio of ATM. Each data point in (**a**,**c**,**d**) represents the mean value of at least five measurements. Standard deviations are indicated by error bars.

The wetting properties of the ATM were checked with a traditional CA measurement. The as-prepared ATM has a CA of 141.6 ± 0.3°, slightly smaller than 150° (Figure 3a). Moreover, the contact angle hysteresis was 8.5°, larger than 5°. Therefore, the ATM is not superhydrophobic [32,33,34]. It is reasonable that the ATM is not superhydrophobic, since a superhydrophobic surface requires micro- and nano-roughness besides the low surface energy of the material [35]. A close look at the contacting interface between the water droplet and the ATM surface revealed that the water droplet sat on top of the micropillars (Figure 3b). We cannot tell whether the water droplet partially penetrated into the gaps among the pillars, due to the technical limitations of our device. However, we would like to assume that the water may have partially penetrated into the gaps, due to gravity. For simplicity, the Cassie equation (2) is used to describe the measured CA:

(2) $${\cos\mathrm{CA}=f}_{1}{\cos\mathrm{CA}}_{i}-f_{2}$$

where *f*_1_ and *f*_2_ = 1 − *f*_1_ represent the fractions of the area of micropillars on ATM contacting the water droplet and the area of air cushions at the interface, respectively. The CA_i_ of PDMS was measured to be 117.4 ± 0.6°. 

While the elongation was increased from 0 to 70%, the CAs were quite close, ranging from 141.6 ± 0.3° to 143.2 ± 0.6° (Figure 3a). It has been argued that the diffuse edge and the uncertainty in the baseline by one pixel could introduce a substantial systematic error in the CA from about 1° to beyond 10° on superhydrophobic surfaces [20]. Here, the change in the CAs is only 1.6°, which means a difference of 1.13%, so that the difference in the CAs could be ignored. 

On the other hand, the air fraction changed significantly upon the mechanical stretching. Without stretching, 1118 ± 26 micropillars were involved in the contact area (Figure 3c), corresponding to an *f*_2_ of 71.99% ± 0.12% (Figure 3d). The increase in elongation to 70% sharply decreased the number of contacted micropillars to 734 ± 30. Accordingly, the *f*_2_ within the contact area increased to 79.01% ± 0.38%. Considering the increase in the air fraction during the sample stretching, we expected an increase of 4.5° in CA, according to the Cassie equation. The accuracy of our device for CA measurement is around 0.01°, which should be far enough to differentiate the difference of 1.6°. However, no clear difference was detected. It implies a drawback in the CA measurement [20]. 

It is well known that the three-phase contact line at the receding front of a water droplet on a tilted surface determines the SA. The water droplet on the ATM has different three-phase contact lines at the receding front, due to the triangular shape of the micropillars when the ATM is tilted in the directions along D_A_ and D_E_. However, no significant difference between the SAs along the opposite directions was detected when P_p_ is 40 µm (corresponding here to the stretching of 0%) [16]. Moreover, no significant difference was found when the period was increased to 51 µm. Though the SA measurement has an accuracy of 0.0001°, which is determined by the electronic tilting base unit, the SA measurement also cannot distinguish the difference between the ATM surfaces under various elongations. 

### 3.3. Friction at Solid-Liquid Interface 

**Figure 4:**
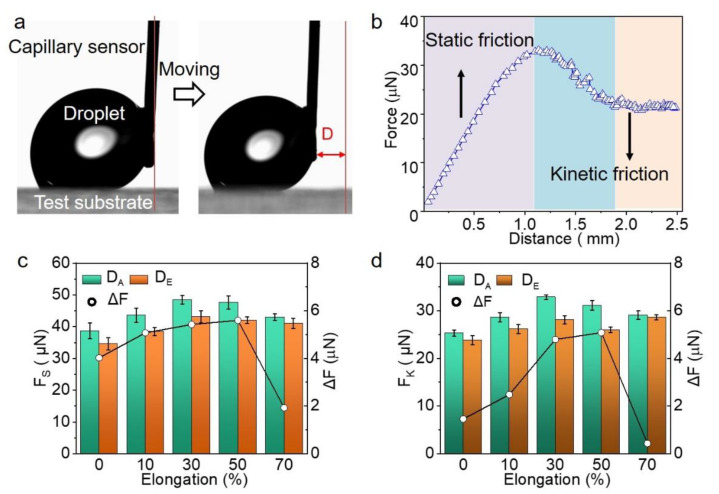
Friction force measurement of a water droplet on ATM. (**a**) Snapshots showing the measurement of friction force; (**b**) Typical curve showing the region of static friction and kinetic friction; (**c**,**d**) The dependence of (**c**) static friction force (F_S_) along D_A_ and D_E_ and their difference (ΔF) and (**d**) kinetic friction force (F_K_) along D_A_ and D_E_ and their difference (ΔF) on the elongation, respectively.

The MPCP technique was used to characterize the ATM surfaces. In a typical measurement, a 6 μL water droplet was dragged by the capillary sensor across the ATM surface. The deflection of the capillary sensor, which caused the resistance between the water droplet and the ATM surface, was converted into friction force (F) (Figure 4a). In analogs to the solid–solid friction, the typical measuring curve can be divided into three regions: static friction; kinetic friction; and the transition zone between the static and the kinetic frictions (Figure 4b) [36]. The peak value during the static friction period was considered as the static friction force (F_S_), while the mean value during the kinetic friction was calculated to be the kinetic friction force (F_K_). On the ATM, the F_S_ of 38.7 ± 2.5 μN and 34.7 ± 1.9 μN were detected when the droplet moved in the direction of the D_A_ and D_E_, respectively (Figure 4c). It means a difference (ΔF) of 4.0 μN, which is around 11.5% in difference along the opposite directions. The stretching to 50% increased the F_S_ in the direction of D_A_ to 47.6 ± 2.6 μN, while in the opposite direction the F_S_ increased to 42.0 ± 1.1 μN. That represents a 23.0% and 21.0% increase in the direction of D_A_ and D_E_, respectively. However, the further increase in the elongation to 70% slightly decreased the F_S_ in the D_A_ and D_E_ directions to 43.0 ± 1.1 and 41.0 ± 1.6 μN, respectively. Accordingly, ΔF also steeply decreased to 1.9 μN, indicating a lower anisotropy of the surface.

Like the solid–solid friction, the F_K_ is smaller than the F_S_ (Figure 4d). For instance, the F_K_s in the direction of the D_A_ and D_E_ were 25.3 ± 0.7 μN and 23.9 ± 1.0 μN, respectively, which are 34.6% and 28.8% smaller than the F_S_. Upon the increase in the elongation, the changes of the F_K_s in both directions are similar to that of the F_S_. That is, the F_K_s in both directions and the corresponding ΔF also reached their maximum at an elongation of 50%, and decreased upon the further stretching to 70%. Therefore, the friction force measurements (F_S_, F_K_, and ΔF) clearly revealed the microscopic anisotropy of the ATM and the structure change of the ATM upon mechanical stretching, showing obvious merits over the measurements of the CA and SA. 

### 3.4. Mechanism for Anisotropic Friction 

**Figure 5:**
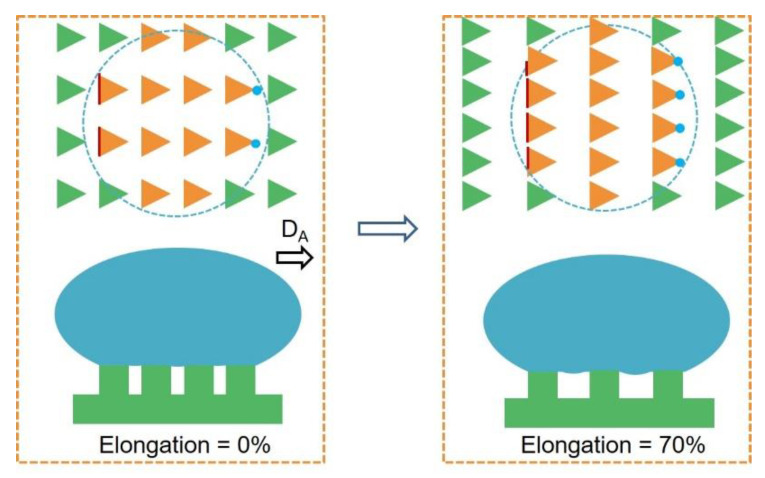
Schematic illustration showing the change in the contact interface between the droplet and ATM at different mechanical stretching. The orange triangles are micropillars in contact with the water droplet and the green triangles are not. The blue dash line indicates the contact perimeter.

The difference in the three-phase contact lines at the receding fronts is responsible for the anisotropic friction (Figure 5). Because of the geometry of the triangular pillar, discrete contact lines (red line) and contact points (blue dots) form at the edge and the angle of the triangular pillars, respectively. When the droplet moves towards D_A_, the receding front is the discrete contact lines. In the opposite direction of D_E_, the discrete contact points locate at the receding front. Since the discrete contact lines provide a large pinning force to the water droplet than the contact points, the friction force along the D_A_ is larger than that along the D_E_ [16]. 

Due to the Poisson’s ratio, the stretching of the ATM (the increase of P_s_) would decrease the space between the micropillars along P_p_, resulting in the increase in the total length of the discrete contact lines and the number of discrete contact points, and, therefore, the friction forces in both directions. However, when the stretching is over 50%, for example, 70%, the increase in the space between the micropillars along P_s_ sharply increases the air fraction at the contact interface; this would dominate the change in the pinning effect of the receding front, reducing the friction forces in both directions and the corresponding ΔF.

### 3.5. Revealing of Surface Periodicity

**Figure 6:**
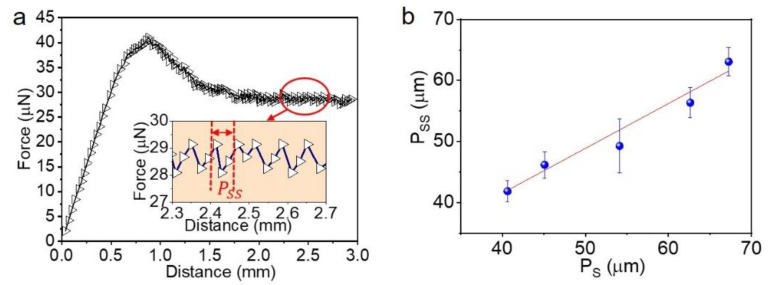
Stick-slip motion during kinetic friction. (**a**) Typical pattern of stick-slip, inset shows the definition of period of stick-slip (P_ss_); (**b**) Dependence of P_ss_ on the period on ATM along the stretching direction (P_S_).

While the measurement of the F_S_ discloses the moment similar to the initiation of sliding of a water droplet on a tilted surface (SA measurement), the process of the F_K_ measurement could reveal the surface structures. On the ATM, the process of kinetic friction showed a stick-slip pattern when the water droplet moved along D_A_ (Figure 6a). While the sticking should be caused by the pinning of the receding front of the droplet, the slipping is assumed to be the result of the receding front jumping from one to the next row of micropillars [37]. The period of the stick-slip (P_SS_) matched the P_S_ quite well at various elongation ratios (Figure 6b). For instance, the P_SS_ revealed a period of 41.86 ± 1.73 μm of the ATM surface at an elongation of 0%, which matches the corresponding P_S_ = 40.62 ± 0.49 μm very well. Upon the increase in the elongation to 70%, the P_S_ and P_SS_ were increased to 67.25 ± 0.26 μm and 63.06 ± 2.31 μm, respectively. It thus strongly suggests the possibility to study the surface geometry by measuring the stick-slip pattern during kinetic friction. 

## 4. Conclusions

In summary, we prepared an array of triangular micropillars (ATM) by standard soft-lithography and examined the wettability by the measurements of CA, SA, and friction force. The CA and SA measurements cannot reveal the microscopic differences in the surface along the opposite directions and the change in the structural parameters upon mechanical stretching. It agrees very well with the concept proposed by Ras, that the contact angle measurement on superhydrophobic surfaces has its inherent drawbacks. The friction measurement conducted by the MPCP technique was involved, in order to examine the ATM surface. The MPCP technique revealed clear differences in the friction forces along the opposite directions and that of the ATM surface under mechanical regulation. Moreover, the kinetic friction process also revealed the surface periodicity, which cannot be achieved by the traditional SA measurement. Increasing the elongation from 0 to 50% increased both the static and kinetic friction forces over 20%, which was attributed to the increased pining effect at the receding fronts. When the elongation reached 70%, however, the friction forces slightly reduced, which was assumed to be caused by the dominating effect of the increased area of the air cushions. We therefore demonstrated the advantage of friction-force measurements in characterizing hydrophobic surfaces over the traditional CA and SA measurements.

## Data Availability

Not applicable.
